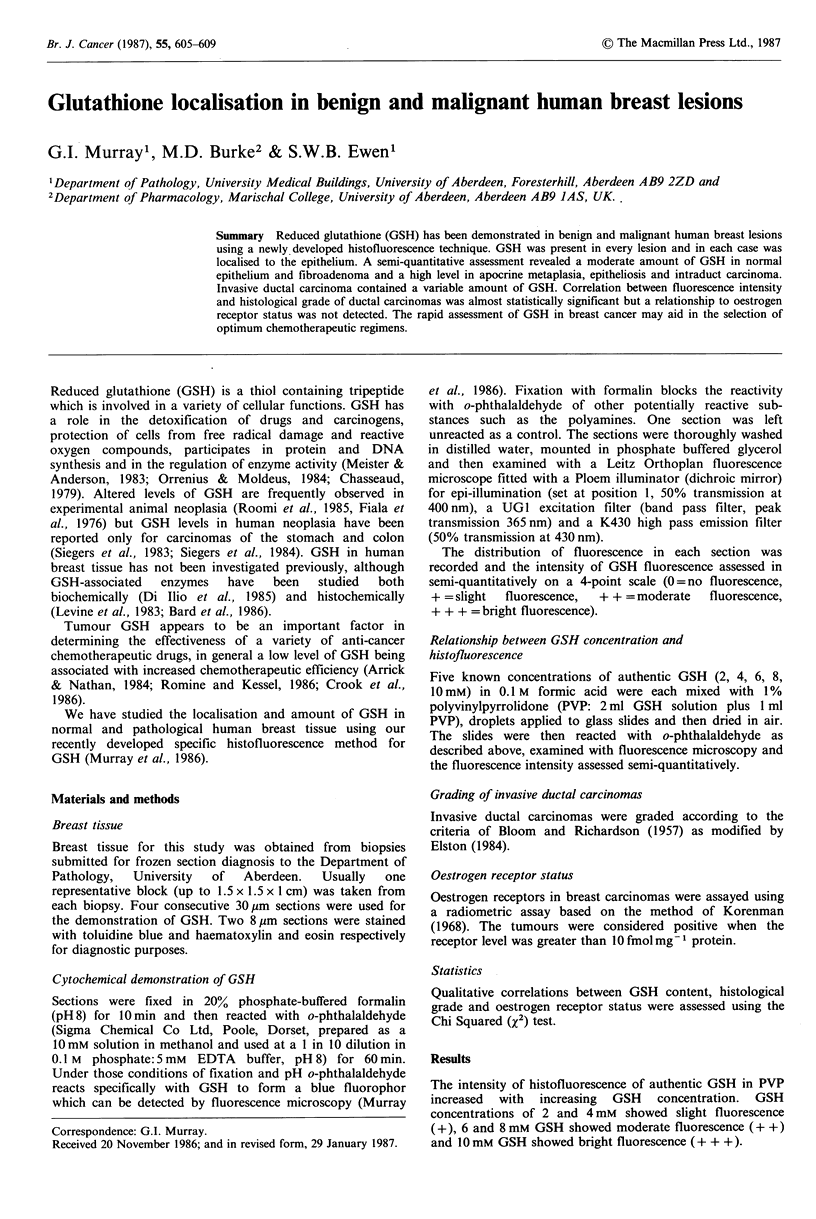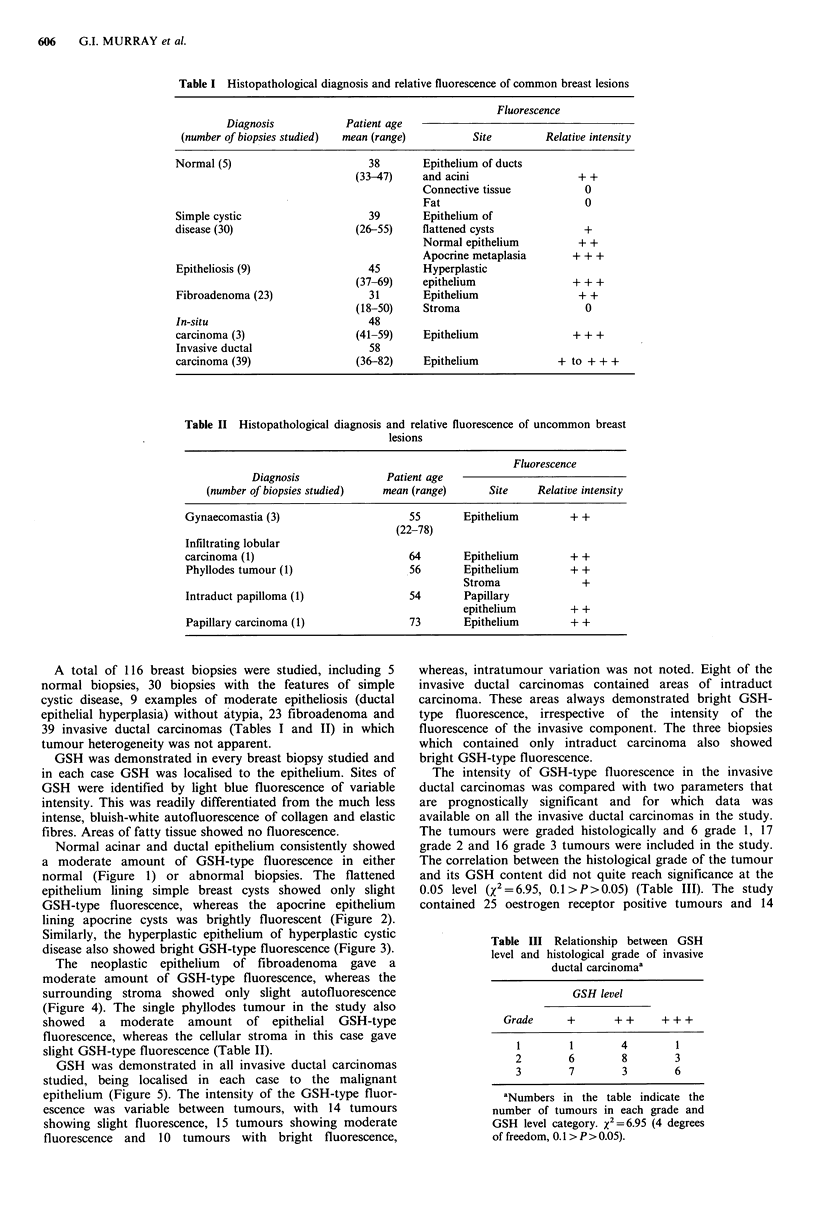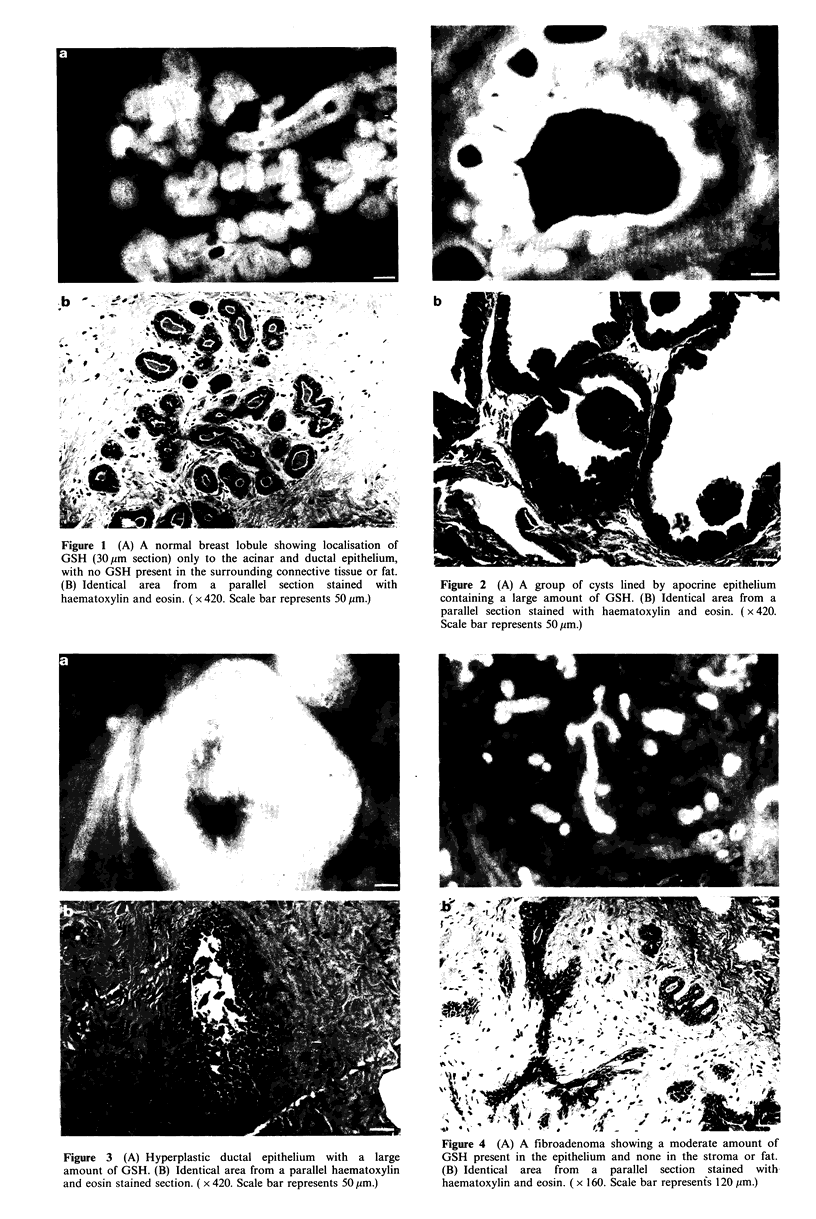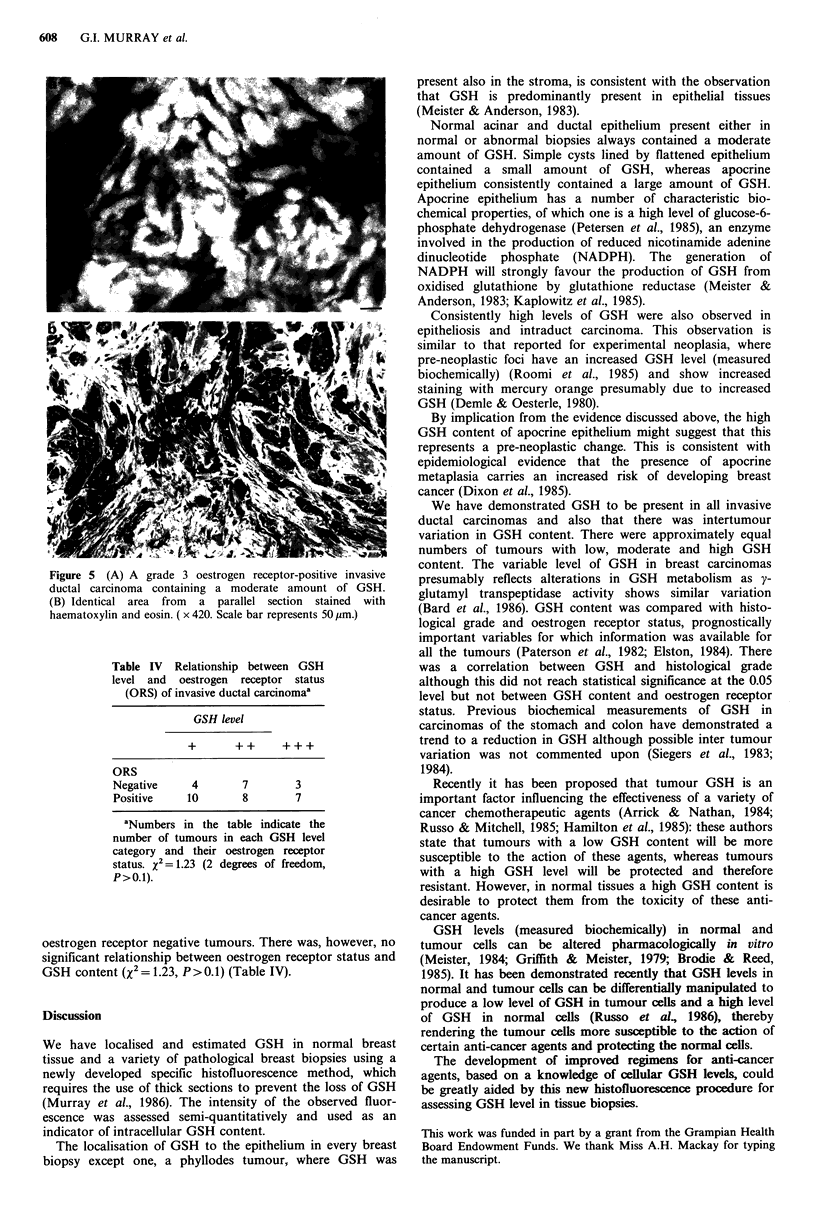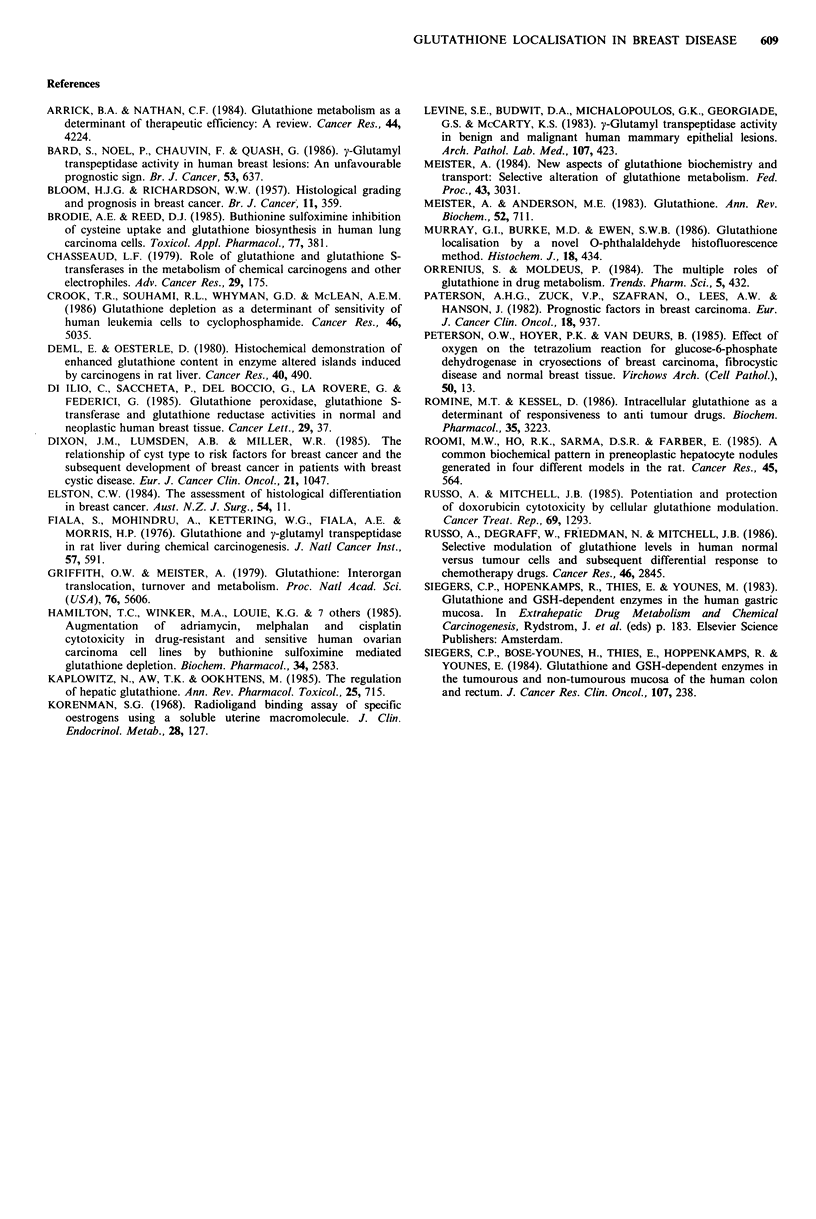# Glutathione localisation in benign and malignant human breast lesions.

**DOI:** 10.1038/bjc.1987.123

**Published:** 1987-06

**Authors:** G. I. Murray, M. D. Burke, S. W. Ewen

## Abstract

**Images:**


Br. J. Cancer (1987), 55, 605–609 © The Macmillan Press Ltd., 1987

### Glutathione localisation in benign and malignant human breast lesions

G.I. Murray[1], M.D. Burke[2] & S.W.B. Ewen[1]

[1]*Department of Pathology, University Medical Buildings, University of Aberdeen, Foresterhill, Aberdeen AB9 2ZD and*
[2]*Department of Pharmacology, Marischal College, University of Aberdeen, Aberdeen AB9 1AS, UK.*

**Summary** Reduced glutathione (GSH) has been demonstrated in benign and malignant human breast lesions using a newly developed histofluorescence technique. GSH was present in every lesion and in each case was localised to the epithelium. A semi-quantitative assessment revealed a moderate amount of GSH in normal epithelium and fibroadenoma and a high level in apocrine metaplasia, epitheliosis and intraduct carcinoma. Invasive ductal carcinoma contained a variable amount of GSH. Correlation between fluorescence intensity and histological grade of ductal carcinomas was almost statistically significant but a relationship to oestrogen receptor status was not detected. The rapid assessment of GSH in breast cancer may aid in the selection of optimum chemotherapeutic regimens.

Reduced glutathione (GSH) is a thiol containing tripeptide which is involved in a variety of cellular functions. GSH has a role in the detoxification of drugs and carcinogens, protection of cells from free radical damage and reactive oxygen compounds, participates in protein and DNA synthesis and in the regulation of enzyme activity (Meister & Anderson, 1983; Orrenius & Moldeus, 1984; Chasseaud, 1979). Altered levels of GSH are frequently observed in experimental animal neoplasia (Roomi *et al.*, 1985, Fiala *et al.*, 1976) but GSH levels in human neoplasia have been reported only for carcinomas of the stomach and colon (Siegers *et al.*, 1983; Siegers *et al.*, 1984). GSH in human breast tissue has not been investigated previously, although GSH-associated enzymes have been studied both biochemically (Di Ilio *et al.*, 1985) and histochemically (Levine *et al.*, 1983; Bard *et al.*, 1986).

Tumour GSH appears to be an important factor in determining the effectiveness of a variety of anti-cancer chemotherapeutic drugs, in general a low level of GSH being associated with increased chemotherapeutic efficiency (Arrick & Nathan, 1984; Romine and Kessel, 1986; Crook *et al.*, 1986).

We have studied the localisation and amount of GSH in normal and pathological human breast tissue using our recently developed specific histofluorescence method for GSH (Murray *et al.*, 1986).

### Materials and methods

### *Breast tissue*

Breast tissue for this study was obtained from biopsies submitted for frozen section diagnosis to the Department of Pathology, University of Aberdeen. Usually one representative block (up to 1.5 × 1.5 × 1 cm) was taken from each biopsy. Four consecutive 30 $\mu$m sections were used for the demonstration of GSH. Two 8 $\mu$m sections were stained with toluidine blue and haematoxylin and eosin respectively for diagnostic purposes.

### *Cytochemical demonstration of GSH*

Sections were fixed in 20% phosphate-buffered formalin (pH 8) for 10 min and then reacted with *o*-phthalaldehyde (Sigma Chemical Co Ltd, Poole, Dorset, prepared as a 10 mM solution in methanol and used at a 1 in 10 dilution in 0.1 M phosphate: 5 mM EDTA buffer, pH 8) for 60 min. Under those conditions of fixation and pH *o*-phthalaldehyde reacts specifically with GSH to form a blue fluorophor which can be detected by fluorescence microscopy (Murray *et al.*, 1986). Fixation with formalin blocks the reactivity with *o*-phthalaldehyde of other potentially reactive substances such as the polyamines. One section was left unreacted as a control. The sections were thoroughly washed in distilled water, mounted in phosphate buffered glycerol and then examined with a Leitz Orthoplan fluorescence microscope fitted with a Ploem illuminator (dichroic mirror) for epi-illumination (set at position 1, 50% transmission at 400 nm), a UG1 excitation filter (band pass filter, peak transmission 365 nm) and a K430 high pass emission filter (50% transmission at 430 nm).

The distribution of fluorescence in each section was recorded and the intensity of GSH fluorescence assessed in semi-quantitatively on a 4-point scale (0 = no fluorescence, + = slight fluorescence, + + = moderate fluorescence, + + + = bright fluorescence).

### *Relationship between GSH concentration and histofluorescence*

Five known concentrations of authentic GSH (2, 4, 6, 8, 10 mM) in 0.1 M formic acid were each mixed with 1% polyvinylpyrrolidone (PVP: 2 ml GSH solution plus 1 ml PVP), droplets applied to glass slides and then dried in air. The slides were then reacted with *o*-phthalaldehyde as described above, examined with fluorescence microscopy and the fluorescence intensity assessed semi-quantitatively.

### *Grading of invasive ductal carcinomas*

Invasive ductal carcinomas were graded according to the criteria of Bloom and Richardson (1957) as modified by Elston (1984).

### *Oestrogen receptor status*

Oestrogen receptors in breast carcinomas were assayed using a radiometric assay based on the method of Korenman (1968). The tumours were considered positive when the receptor level was greater than 10 fmol mg$^{-1}$ protein.

### *Statistics*

Qualitative correlations between GSH content, histological grade and oestrogen receptor status were assessed using the Chi Squared ($\chi^2$) test.

### Results

The intensity of histofluorescence of authentic GSH in PVP increased with increasing GSH concentration. GSH concentrations of 2 and 4 mM showed slight fluorescence (+), 6 and 8 mM GSH showed moderate fluorescence (+ +) and 10 mM GSH showed bright fluorescence (+ + +).

Correspondence: G.I. Murray.
Received 20 November 1986; and in revised form, 29 January 1987.

**Table I** Histopathological diagnosis and relative fluorescence of common breast lesions

| Diagnosis (number of biopsies studied) | Patient age mean (range) | Fluorescence Site | Relative intensity |
|---|---|---|---|
| Normal (5) | 38 (33–47) | Epithelium of ducts and acini | ++ |
| | | Connective tissue | 0 |
| | | Fat | 0 |
| Simple cystic disease (30) | 39 (26–55) | Epithelium of flattened cysts | + |
| | | Normal epithelium | ++ |
| | | Apocrine metaplasia | +++ |
| Epitheliosis (9) | 45 (37–69) | Hyperplastic epithelium | +++ |
| Fibroadenoma (23) | 31 (18–50) | Epithelium | ++ |
| | | Stroma | 0 |
| *In-situ* carcinoma (3) | 48 (41–59) | Epithelium | +++ |
| Invasive ductal carcinoma (39) | 58 (36–82) | Epithelium | + to +++ |

**Table II** Histopathological diagnosis and relative fluorescence of uncommon breast lesions

| Diagnosis (number of biopsies studied) | Patient age mean (range) | Fluorescence Site | Relative intensity |
|---|---|---|---|
| Gynaecomastia (3) | 55 (22–78) | Epithelium | ++ |
| Infiltrating lobular carcinoma (1) | 64 | Epithelium | ++ |
| Phyllodes tumour (1) | 56 | Epithelium | ++ |
| | | Stroma | + |
| Intraduct papilloma (1) | 54 | Papillary epithelium | ++ |
| Papillary carcinoma (1) | 73 | Epithelium | ++ |

A total of 116 breast biopsies were studied, including 5 normal biopsies, 30 biopsies with the features of simple cystic disease, 9 examples of moderate epitheliosis (ductal epithelial hyperplasia) without atypia, 23 fibroadenoma and 39 invasive ductal carcinomas (Tables I and II) in which tumour heterogeneity was not apparent.

GSH was demonstrated in every breast biopsy studied and in each case GSH was localised to the epithelium. Sites of GSH were identified by light blue fluorescence of variable intensity. This was readily differentiated from the much less intense, bluish-white autofluorescence of collagen and elastic fibres. Areas of fatty tissue showed no fluorescence.

Normal acinar and ductal epithelium consistently showed a moderate amount of GSH-type fluorescence in either normal (Figure 1) or abnormal biopsies. The flattened epithelium lining simple breast cysts showed only slight GSH-type fluorescence, whereas the apocrine epithelium lining apocrine cysts was brightly fluorescent (Figure 2). Similarly, the hyperplastic epithelium of hyperplastic cystic disease also showed bright GSH-type fluorescence (Figure 3).

The neoplastic epithelium of fibroadenoma gave a moderate amount of GSH-type fluorescence, whereas the surrounding stroma showed only slight autofluorescence (Figure 4). The single phyllodes tumour in the study also showed a moderate amount of epithelial GSH-type fluorescence, whereas the cellular stroma in this case gave slight GSH-type fluorescence (Table II).

GSH was demonstrated in all invasive ductal carcinomas studied, being localised in each case to the malignant epithelium (Figure 5). The intensity of the GSH-type fluorescence was variable between tumours, with 14 tumours showing slight fluorescence, 15 tumours showing moderate fluorescence and 10 tumours with bright fluorescence, whereas, intratumour variation was not noted. Eight of the invasive ductal carcinomas contained areas of intraduct carcinoma. These areas always demonstrated bright GSH-type fluorescence, irrespective of the intensity of the fluorescence of the invasive component. The three biopsies which contained only intraduct carcinoma also showed bright GSH-type fluorescence.

The intensity of GSH-type fluorescence in the invasive ductal carcinomas was compared with two parameters that are prognostically significant and for which data was available on all the invasive ductal carcinomas in the study. The tumours were graded histologically and 6 grade 1, 17 grade 2 and 16 grade 3 tumours were included in the study. The correlation between the histological grade of the tumour and its GSH content did not quite reach significance at the 0.05 level ($\chi^2=6.95$, $0.1>P>0.05$) (Table III). The study contained 25 oestrogen receptor positive tumours and 14

**Table III** Relationship between GSH level and histological grade of invasive ductal carcinoma[a]

| Grade | GSH level + | ++ | +++ |
|---|---|---|---|
| 1 | 1 | 4 | 1 |
| 2 | 6 | 8 | 3 |
| 3 | 7 | 3 | 6 |

[a]Numbers in the table indicate the number of tumours in each grade and GSH level category. $\chi^2=6.95$ (4 degrees of freedom, $0.1>P>0.05$).

**Figure 1** (A) A normal breast lobule showing localisation of GSH (30 μm section) only to the acinar and ductal epithelium, with no GSH present in the surrounding connective tissue or fat. (B) Identical area from a parallel section stained with haematoxylin and eosin. ( × 420. Scale bar represents 50 μm.)

**Figure 2** (A) A group of cysts lined by apocrine epithelium containing a large amount of GSH. (B) Identical area from a parallel section stained with haematoxylin and eosin. ( × 420. Scale bar represents 50 μm.)

**Figure 3** (A) Hyperplastic ductal epithelium with a large amount of GSH. (B) Identical area from a parallel haematoxylin and eosin stained section. ( × 420. Scale bar represents 50 μm.)

**Figure 4** (A) A fibroadenoma showing a moderate amount of GSH present in the epithelium and none in the stroma or fat. (B) Identical area from a parallel section stained with haematoxylin and eosin. ( × 160. Scale bar represents 120 μm.)

**Figure 5** (A) A grade 3 oestrogen receptor-positive invasive ductal carcinoma containing a moderate amount of GSH. (B) Identical area from a parallel section stained with haematoxylin and eosin. ( × 420. Scale bar represents 50 $\mu$m.)

**Table IV** Relationship between GSH level and oestrogen receptor status (ORS) of invasive ductal carcinoma[a]

| | *GSH level* | | |
|---|---|---|---|
| | + | ++ | +++ |
| ORS | | | |
| Negative | 4 | 7 | 3 |
| Positive | 10 | 8 | 7 |

[a]Numbers in the table indicate the number of tumours in each GSH level category and their oestrogen receptor status. $\chi^2 = 1.23$ (2 degrees of freedom, $P > 0.1$).

oestrogen receptor negative tumours. There was, however, no significant relationship between oestrogen receptor status and GSH content ($\chi^2 = 1.23$, $P > 0.1$) (Table IV).

### Discussion

We have localised and estimated GSH in normal breast tissue and a variety of pathological breast biopsies using a newly developed specific histofluorescence method, which requires the use of thick sections to prevent the loss of GSH (Murray *et al.*, 1986). The intensity of the observed fluorescence was assessed semi-quantitatively and used as an indicator of intracellular GSH content.

The localisation of GSH to the epithelium in every breast biopsy except one, a phyllodes tumour, where GSH was present also in the stroma, is consistent with the observation that GSH is predominantly present in epithelial tissues (Meister & Anderson, 1983).

Normal acinar and ductal epithelium present either in normal or abnormal biopsies always contained a moderate amount of GSH. Simple cysts lined by flattened epithelium contained a small amount of GSH, whereas apocrine epithelium consistently contained a large amount of GSH. Apocrine epithelium has a number of characteristic biochemical properties, of which one is a high level of glucose-6-phosphate dehydrogenase (Petersen *et al.*, 1985), an enzyme involved in the production of reduced nicotinamide adenine dinucleotide phosphate (NADPH). The generation of NADPH will strongly favour the production of GSH from oxidised glutathione by glutathione reductase (Meister & Anderson, 1983; Kaplowitz *et al.*, 1985).

Consistently high levels of GSH were also observed in epitheliosis and intraduct carcinoma. This observation is similar to that reported for experimental neoplasia, where pre-neoplastic foci have an increased GSH level (measured biochemically) (Roomi *et al.*, 1985) and show increased staining with mercury orange presumably due to increased GSH (Demle & Oesterle, 1980).

By implication from the evidence discussed above, the high GSH content of apocrine epithelium might suggest that this represents a pre-neoplastic change. This is consistent with epidemiological evidence that the presence of apocrine metaplasia carries an increased risk of developing breast cancer (Dixon *et al.*, 1985).

We have demonstrated GSH to be present in all invasive ductal carcinomas and also that there was intertumour variation in GSH content. There were approximately equal numbers of tumours with low, moderate and high GSH content. The variable level of GSH in breast carcinomas presumably reflects alterations in GSH metabolism as $\gamma$-glutamyl transpeptidase activity shows similar variation (Bard *et al.*, 1986). GSH content was compared with histological grade and oestrogen receptor status, prognostically important variables for which information was available for all the tumours (Paterson *et al.*, 1982; Elston, 1984). There was a correlation between GSH and histological grade although this did not reach statistical significance at the 0.05 level but not between GSH content and oestrogen receptor status. Previous biochemical measurements of GSH in carcinomas of the stomach and colon have demonstrated a trend to a reduction in GSH although possible inter tumour variation was not commented upon (Siegers *et al.*, 1983; 1984).

Recently it has been proposed that tumour GSH is an important factor influencing the effectiveness of a variety of cancer chemotherapeutic agents (Arrick & Nathan, 1984; Russo & Mitchell, 1985; Hamilton *et al.*, 1985): these authors state that tumours with a low GSH content will be more susceptible to the action of these agents, whereas tumours with a high GSH level will be protected and therefore resistant. However, in normal tissues a high GSH content is desirable to protect them from the toxicity of these anti-cancer agents.

GSH levels (measured biochemically) in normal and tumour cells can be altered pharmacologically *in vitro* (Meister, 1984; Griffith & Meister, 1979; Brodie & Reed, 1985). It has been demonstrated recently that GSH levels in normal and tumour cells can be differentially manipulated to produce a low level of GSH in tumour cells and a high level of GSH in normal cells (Russo *et al.*, 1986), thereby rendering the tumour cells more susceptible to the action of certain anti-cancer agents and protecting the normal cells.

The development of improved regimens for anti-cancer agents, based on a knowledge of cellular GSH levels, could be greatly aided by this new histofluorescence procedure for assessing GSH level in tissue biopsies.

This work was funded in part by a grant from the Grampian Health Board Endowment Funds. We thank Miss A.H. Mackay for typing the manuscript.

### References

ARRICK, B.A. & NATHAN, C.F. (1984). Glutathione metabolism as a determinant of therapeutic efficiency: A review. *Cancer Res.*, **44**, 4224.

BARD, S., NOEL, P., CHAUVIN, F. & QUASH, G. (1986). $\gamma$-Glutamyl transpeptidase activity in human breast lesions: An unfavourable prognostic sign. *Br. J. Cancer*, **53**, 637.

BLOOM, H.J.G. & RICHARDSON, W.W. (1957). Histological grading and prognosis in breast cancer. *Br. J. Cancer*, **11**, 359.

BRODIE, A.E. & REED, D.J. (1985). Buthionine sulfoximine inhibition of cysteine uptake and glutathione biosynthesis in human lung carcinoma cells. *Toxicol. Appl. Pharmacol.*, **77**, 381.

CHASSEAUD, L.F. (1979). Role of glutathione and glutathione S-transferases in the metabolism of chemical carcinogens and other electrophiles. *Adv. Cancer Res.*, **29**, 175.

CROOK, T.R., SOUHAMI, R.L., WHYMAN, G.D. & McLEAN, A.E.M. (1986) Glutathione depletion as a determinant of sensitivity of human leukemia cells to cyclophosphamide. *Cancer Res.*, **46**, 5035.

DEML, E. & OESTERLE, D. (1980). Histochemical demonstration of enhanced glutathione content in enzyme altered islands induced by carcinogens in rat liver. *Cancer Res.*, **40**, 490.

DI ILIO, C., SACCHETA, P., DEL BOCCIO, G., LA ROVERE, G. & FEDERICI, G. (1985). Glutathione peroxidase, glutathione S-transferase and glutathione reductase activities in normal and neoplastic human breast tissue. *Cancer Lett.*, **29**, 37.

DIXON, J.M., LUMSDEN, A.B. & MILLER, W.R. (1985). The relationship of cyst type to risk factors for breast cancer and the subsequent development of breast cancer in patients with breast cystic disease. *Eur. J. Cancer Clin. Oncol.*, **21**, 1047.

ELSTON, C.W. (1984). The assessment of histological differentiation in breast cancer. *Aust. N.Z. J. Surg.*, **54**, 11.

FIALA, S., MOHINDRU, A., KETTERING, W.G., FIALA, A.E. & MORRIS, H.P. (1976). Glutathione and $\gamma$-glutamyl transpeptidase in rat liver during chemical carcinogenesis. *J. Natl Cancer Inst.*, **57**, 591.

GRIFFITH, O.W. & MEISTER, A. (1979). Glutathione: Interorgan translocation, turnover and metabolism. *Proc. Natl Acad. Sci.* (*USA*), **76**, 5606.

HAMILTON, T.C., WINKER, M.A., LOUIE, K.G. & 7 others (1985). Augmentation of adriamycin, melphalan and cisplatin cytotoxicity in drug-resistant and sensitive human ovarian carcinoma cell lines by buthionine sulfoximine mediated glutathione depletion. *Biochem. Pharmacol.*, **34**, 2583.

KAPLOWITZ, N., AW, T.K. & OOKHTENS, M. (1985). The regulation of hepatic glutathione. *Ann. Rev. Pharmacol. Toxicol.*, **25**, 715.

KORENMAN, S.G. (1968). Radioligand binding assay of specific oestrogens using a soluble uterine macromolecule. *J. Clin. Endocrinol. Metab.*, **28**, 127.

LEVINE, S.E., BUDWIT, D.A., MICHALOPOULOS, G.K., GEORGIADE, G.S. & McCARTY, K.S. (1983). $\gamma$-Glutamyl transpeptidase activity in benign and malignant human mammary epithelial lesions. *Arch. Pathol. Lab. Med.*, **107**, 423.

MEISTER, A. (1984). New aspects of glutathione biochemistry and transport: Selective alteration of glutathione metabolism. *Fed. Proc.*, **43**, 3031.

MEISTER, A. & ANDERSON, M.E. (1983). Glutathione. *Ann. Rev. Biochem.*, **52**, 711.

MURRAY, G.I., BURKE, M.D. & EWEN, S.W.B. (1986). Glutathione localisation by a novel O-phthalaldehyde histofluorescence method. *Histochem. J.*, **18**, 434.

ORRENIUS, S. & MOLDEUS, P. (1984). The multiple roles of glutathione in drug metabolism. *Trends. Pharm. Sci.*, **5**, 432.

PATERSON, A.H.G., ZUCK, V.P., SZAFRAN, O., LEES, A.W. & HANSON, J. (1982). Prognostic factors in breast carcinoma. *Eur. J. Cancer Clin. Oncol.*, **18**, 937.

PETERSON, O.W., HOYER, P.K. & VAN DEURS, B. (1985). Effect of oxygen on the tetrazolium reaction for glucose-6-phosphate dehydrogenase in cryosections of breast carcinoma, fibrocystic disease and normal breast tissue. *Virchows Arch.* (*Cell Pathol.*), **50**, 13.

ROMINE, M.T. & KESSEL, D. (1986). Intracellular glutathione as a determinant of responsiveness to anti tumour drugs. *Biochem. Pharmacol.*, **35**, 3223.

ROOMI, M.W., HO, R.K., SARMA, D.S.R. & FARBER, E. (1985). A common biochemical pattern in preneoplastic hepatocyte nodules generated in four different models in the rat. *Cancer Res.*, **45**, 564.

RUSSO, A. & MITCHELL, J.B. (1985). Potentiation and protection of doxorubicin cytotoxicity by cellular glutathione modulation. *Cancer Treat. Rep.*, **69**, 1293.

RUSSO, A., DEGRAFF, W., FRIEDMAN, N. & MITCHELL, J.B. (1986). Selective modulation of glutathione levels in human normal versus tumour cells and subsequent differential response to chemotherapy drugs. *Cancer Res.*, **46**, 2845.

SIEGERS, C.P., HOPENKAMPS, R., THIES, E. & YOUNES, M. (1983). Glutathione and GSH-dependent enzymes in the human gastric mucosa. In *Extrahepatic Drug Metabolism and Chemical Carcinogenesis*, Rydstrom, J. *et al.* (eds) p. 183. Elsevier Science Publishers: Amsterdam.

SIEGERS, C.P., BOSE-YOUNES, H., THIES, E., HOPPENKAMPS, R. & YOUNES, E. (1984). Glutathione and GSH-dependent enzymes in the tumourous and non-tumourous mucosa of the human colon and rectum. *J. Cancer Res. Clin. Oncol.*, **107**, 238.